# Cambinol, a Novel Inhibitor of Neutral Sphingomyelinase 2 Shows Neuroprotective Properties

**DOI:** 10.1371/journal.pone.0124481

**Published:** 2015-05-26

**Authors:** Mariana Figuera-Losada, Marigo Stathis, Joelle M. Dorskind, Ajit G. Thomas, Veera Venkata Ratnam Bandaru, Seung-Wan Yoo, Nicholas J. Westwood, Graeme W. Rogers, Justin C. McArthur, Norman J. Haughey, Barbara S. Slusher, Camilo Rojas

**Affiliations:** 1 Brain Science Institute Drug Discovery Program, Johns Hopkins University School of Medicine, Baltimore, Maryland, United States of America; 2 Department of Neurology, Johns Hopkins University School of Medicine, Baltimore, Maryland, United States of America; 3 School of Chemistry and Biomedical Sciences Research Centre, University of Saint Andrews and EaStCHEM, North Haugh, Saint Andrews, Fife, KY16 9ST, United Kingdom; 4 Department of Psychiatry, Johns Hopkins University School of Medicine, Baltimore, Maryland, United States of America; 5 Department of Neuroscience, Johns Hopkins University School of Medicine, Baltimore, Maryland, United States of America; 6 Department of Molecular and Comparative Pathobiology, Johns Hopkins University School of Medicine, Baltimore, Maryland, United States of America; 7 Richard T. Johnson Division of Neuroimmunology and Neurological Infections, Johns Hopkins University School of Medicine, Baltimore, Maryland, United States of America; 8 Johns Hopkins University School of Medicine, Baltimore, Maryland, United States of America

## Abstract

Ceramide is a bioactive lipid that plays an important role in stress responses leading to apoptosis, cell growth arrest and differentiation. Ceramide production is due in part to sphingomyelin hydrolysis by sphingomyelinases. In brain, neutral sphingomyelinase 2 (nSMase2) is expressed in neurons and increases in its activity and expression have been associated with pro-inflammatory conditions observed in Alzheimer’s disease, multiple sclerosis and human immunodeficiency virus (HIV-1) patients. Increased nSMase2 activity translates into higher ceramide levels and neuronal cell death, which can be prevented by chemical or genetic inhibition of nSMase2 activity or expression. However, to date, there are no soluble, specific and potent small molecule inhibitor tool compounds for *in vivo* studies or as a starting point for medicinal chemistry optimization. Moreover, the majority of the known inhibitors were identified using bacterial, bovine or rat nSMase2. In an attempt to identify new inhibitor scaffolds, two activity assays were optimized as screening platform using the recombinant human enzyme. First, active hits were identified using a fluorescence-based high throughput compatible assay. Then, hits were confirmed using a ^14^C sphingomyelin-based direct activity assay. Pharmacologically active compounds and approved drugs were screened using this strategy which led to the identification of cambinol as a novel uncompetitive nSMase2 inhibitor (*K*_i_ = 7 μM). The inhibitory activity of cambinol for nSMase2 was approximately 10-fold more potent than for its previously known target, silence information regulator 1 and 2 (SIRT1/2). Cambinol decreased tumor necrosis factor-α or interleukin-1 β-induced increases of ceramide and cell death in primary neurons. A preliminary study of cambinol structure and activity allowed the identification of the main structural features required for nSMase2 inhibition. Cambinol and its analogs may be useful as nSMase2 inhibitor tool compounds to prevent ceramide-dependent neurodegeneration.

## Introduction

**Fig 1:**
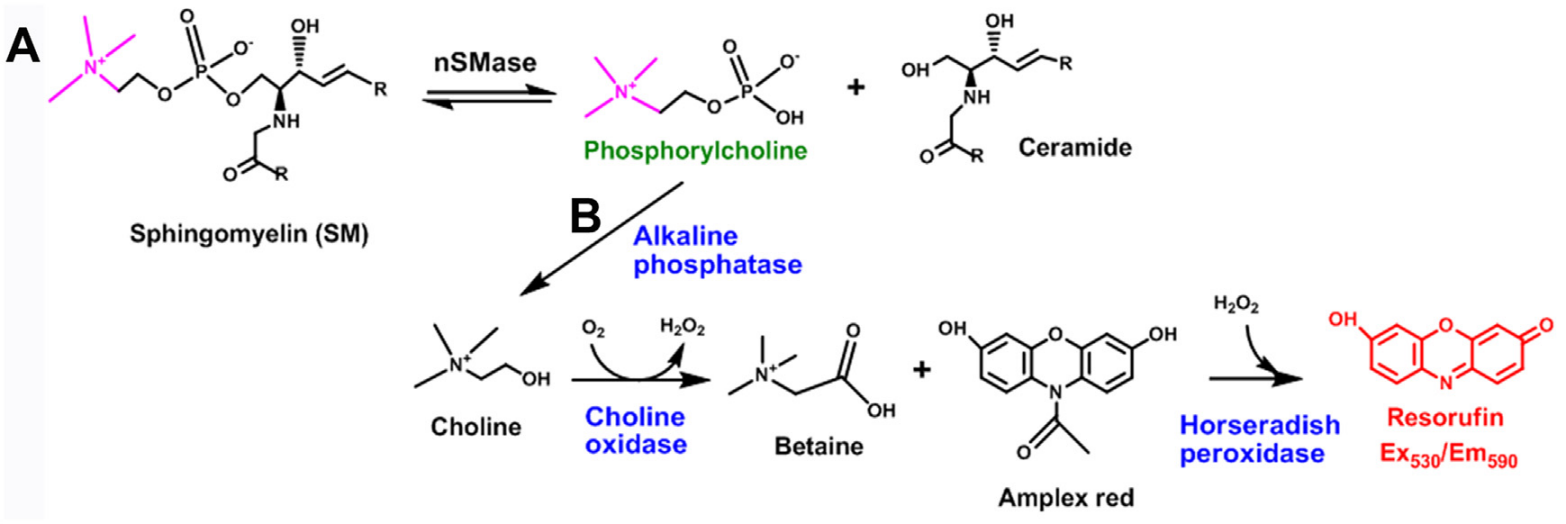
Fluorescence and radioactivity assays for human nSMase2. **(A)** nSMase2-catalyzed reaction in the direct confirmatory assay with ^14^C-labeled SM (labeled atoms shown in magenta), which is hydrolyzed to generate labeled product. **(B)** Fluorescence assay for human nSMase2 consisting of three coupled reactions (in blue) and Amplex red dye to generate a red fluorescent product, resorufin (in red).

Sphingolipids are a major component of eukaryotic cell membranes. They are enriched in lipid rafts, and are particularly abundant in the nervous systems of mammals [1]. Ceramides are a class of sphingolipid that play structural roles in biological membranes and regulate a variety of cellular functions such as apoptosis, autophagy, proliferation, cell adhesion, differentiation, migration, senescence and intracellular trafficking [1,2]. Although several pathways are responsible for the generation of ceramide, the hydrolysis of sphingomyelin (SM) to produce ceramide and phosphorylcholine (Fig 1A) appears to be prevalent during responses to stress [3,4]. This reaction is catalyzed by a family of sphingomyelin phosphodiesterase enzymes, also known as sphingomyelinases (E.C. 3.1.4.12). To date, four neutral sphingomyelinases (nSMase) have been identified: nSMase1, nSMase2, nSMase3 and mitochondria-associated nSMase; a lysosomal acid sphingomyelinase (aSMase) and an alkaline sphingomyelinase [3,4]. These enzymes differ in primary structure, subcellular localization, tissue distribution, pH optima and metal-dependence for activity. Human nSMase2 requires neutral pH and Mg^2+^ ions for activity. It localizes to the inner leaflet of the plasma membrane and the Golgi apparatus [3,5] and it is the predominant sphingomyelinase in the brain [6].

Ceramide production occurs in response to reactive oxygen species, pro-inflammatory stimuli, HIV-1 proteins, amyloid beta (Aβ), radiation, and chemotherapeutic agents through induction of nSMase2 [7–10]. Dysregulation of ceramide metabolism has been reported in HIV-associated neurocognitive disorders (HAND), Alzheimer’s disease (AD), Parkinson’s disease (PD), amyotrophic lateral sclerosis (ALS), and chronic inflammation [11–16]. With respect to HAND, Bandaru *et al*. [12] found increased ceramide and human nSMase2 levels in the cerebrospinal fluid of patients with HIV-associated dementia as compared to control individual with no cognitive impairments. *In vitro*, HIV-1 gp120 and Tat proteins induced ceramide production, altered mitochondrial function and increased oxidative stress [17]. In AD, postmortem analysis of the brains of patients showed increased ceramide levels, oxidative stress and inflammation [18,19] and blood ceramides have been associated with progression of cognitive impairment [20–22]. Additionally, treatment of oligodendrocytes or neurons with Aβ peptide caused SM hydrolysis and ceramide accumulation presumably through the actions of a nSMase [19,23]. Together these data suggest that accumulation of ceramide through the actions of nSMase is associated with neural cell damage in a variety of disease settings.

Recently, the activity of nSMase2 and a ceramide-dependent process have been linked to the release of exosomes [24]. These are membranous particles or vesicles between 20–100 nm that transport cargo between cells and play an important role in intercellular communication [25]. Evidence suggests that exosomes play a role in pathologies such as cancer, HIV-1 infection, prion disease, PD and AD harboring pro-apoptotic signals, cytokines, adhesion proteins, antigen presenting receptors, antigens, RNA and viral particles [26–32].

Inhibition of human nSMase2 may preserve neuronal function by preventing increases in ceramide, and/or blocking exosome production and as a consequence decreasing cell death. Pharmacological inhibition of nSMase2 activity or molecular interference have been shown to protect neurons, oligodendrocytes and astrocytes against ceramide induced-cell death produced by gp120, Tat, TNF-α, IL-1β, Aβ and ischemia [7,13,15,33–40].

**Fig 2:**
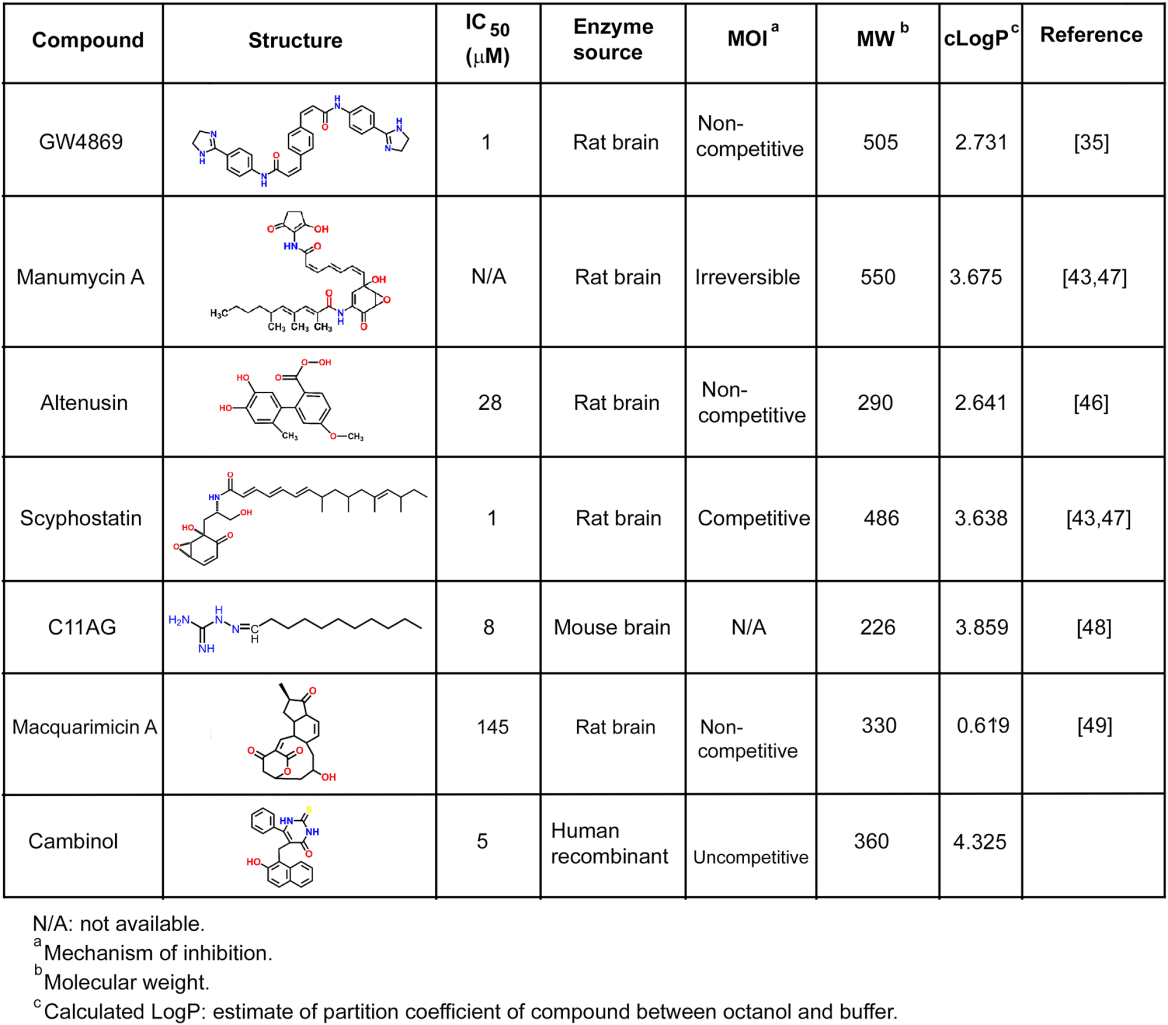
Prototype nSMase inhibitors. Table showing the most well-known nSMase inhibitors, their potency, the species of nSMase in which the compounds were first identified and their physicochemical properties.

To date, a number of nSMase inhibitors have been identified, but these compounds have molecular characteristics that make them poor drug candidates including a lack of specificity, low potency, and undesirable physicochemical properties that reduce drug-likeness and blood-brain barrier penetration [41,42] (Fig 2). Among the synthetic molecules and natural products that have been described as nSMase inhibitors, GW4869 [35] is the most well characterized and used prototype inhibitor. However, this compound is poorly soluble (0.2 mg/ml in DMSO) and lacks drug-like molecular properties. Additionally, many of the known nSMase inhibitors were identified using bacterial, bovine or rat enzyme rather than the human form. These non-human forms of nSMase2 contain considerable sequence and modulatory site differences [43–46].

In an attempt to find more drug-like nSMase2 inhibitors we optimized two biochemical assays using recombinant human enzyme with respect to time, substrate and enzyme concentrations. One assay consisted of monitoring human nSMase2 activity in 384-well format using three sequential coupled reactions that generate a fluorescent product. The other assay involved the use of ^14^C-labeled SM to directly follow human nSMase2 activity by quantifying the radioactive product. These assays were used as primary and confirmatory assays, respectively, to screen two focused libraries of pharmacologically active compounds. From this screen, cambinol was identified as an uncompetitive inhibitor of human nSMase2 with a *K*_i_ value of 7 μM (IC_50_ = 5 ± 1 μM). Subsequent studies revealed that cambinol could dose dependently block TNF-α-induced increase in ceramide and prevent TNF-α or IL-1β-induced cell death or dendritic damage in rat primary neurons demonstrating the potential of the newly developed screening methods to identify novel human nSMase2 inhibitors.

## Materials and Methods

### Expression of human nSMase2

Full length human nSMase2 cDNA with a C-terminal Flag tag cloned in pCMV6-Entry expression vector (Origene) was transfected into HEK293 cells using lipofectamine 2000 (Life Technologies). Selection of transfected cells was carried out for two weeks with 500 μg/ml G418 in EMEM containing 10% FBS (ATCC) and 2 mM glutamine (Life Technologies). Expression of human nSMase2 was confirmed by Western-blot analysis using an antibody specific against the protein (R&D) diluted to 0.4 μg/ml in Tris-buffered saline with 0.1% Tween 20 and 5% bovine serum albumin.

Cells stably and constitutively expressing human nSMase2 were grown to confluency in 150 mm dishes, washed twice with cold PBS and harvested using a cell scraper in 1 ml of lysis buffer per plate [100 mM Tris-HCl pH 7.5, 1 mM EDTA, 100 mM sucrose, 100 μM PMSF, 1X protease inhibitor cocktail III (Calbiochem)]. Cell lysis was achieved by sonicating 3 times on ice for 30 sec. Cell lysate protein concentration was determined by bicinchoninic acid (BCA) assay. Aliquots of cell lysate were snap frozen and stored at -80°C. Activity of the recombinant enzyme remained stable for at least six months.

### Human nSMase2 activity assays

#### Fluorescence assay

To monitor the activity of human nSMase2, lysate of cells expressing the recombinant enzyme (0.5–1 μg protein per reaction) was used to catalyze the hydrolysis of SM (250 μM) into ceramide and phosphorylcholine. The production of the latter was coupled to a dephosphorylation reaction catalyzed by alkaline phosphatase (4 U/ml) to produce choline, followed by oxidation of choline by choline oxidase (0.1 U/ml) to produce betaine and H_2_O_2_, which in the presence of horseradish peroxidase (HRP, 1U/ml) and 50 μM Amplex red, generates the fluorescent molecule resorufin (Life Technologies). Substrate stock solution was prepared in 2% Triton X-100, vortexed and sonicated for 30 min. Reactions were carried out for 1 h at 37°C in 100 mM Tris-HCl pH 7.4, 10 mM MgCl_2_, 0.2% Triton X-100 and the generation of the fluorescent product was monitored by measuring relative florescence units (RFU) with excitation at 530 nm and emission at 590 nm (Fig 1). This assay was optimized for 384-well format (50 μl total volume per well) based on linearity of the enzymatic activity with respect to time, as well as substrate and enzyme concentrations. Human nSMase2 Mg^2+^-dependence was confirmed by adding 50 mM EDTA to the reaction buffer in order to chelate the ion. This fluorescence assay was used for compound screening and IC_50_ value determinations, based on duplicate eight-point dose response curves. Identification of inhibitors other than competitive was favored given that the concentration of SM used was higher than the experimentally determined *K*_m_ value.

As a counter screening assay, the alkaline phosphatase, choline oxidase and HRP reactions were carried out in the absence of human nSMase2 and SM. To initiate reactions, the alkaline phosphatase substrate, phosphorylcholine, was provided at a concentration of 16 μM, which was found to provide optimal stability over time and signal to noise ratio. Compounds that showed inhibitory activity in this assay were characterized no further. Results are representative of at least two independent experiments run in duplicate. Data analysis and non-linear least squares curve fitting were done with GraphPad Prism 5 and Grafit.

#### ^14^C-SM confirmatory assay

This assay was dependent on the direct hydrolysis of ^14^C-labeled SM (^14^C N-methyl groups in the phosphorylcholine) by human nSMase2. Optimization of assay conditions was accomplished according to the methods of Barbone *et al*. [47]. Briefly, bovine [N-methyl-^14^C]-sphingomyelin (specific activity 52 mCi/mol, Perkin Elmer) and phosphatidylinositol (Avanti Polar Lipids) were evaporated to dryness under a stream of nitrogen and resuspended at an equimolar concentration of 0.1 mM in water with 0.5% Triton X-100 (Sigma). The solution was vortexed for 30 min and then sonicated for 30 min. Assay conditions were as follows: 50 μl total volume per reaction, consisting of 20 μM SM, reaction buffer (100 mM Tris-HCl pH 7.5, 80 mM MgCl_2_, 0.1% Triton X-100); 10 μl cell lysate containing recombinant human nSMase2 (0.5–1 μg protein/assay) and 5 μl of compound in reaction buffer containing 10% DMSO. After 1 h incubation at 37°C the reaction was terminated by the addition of 30 μl of water and 175 μl of chloroform/methanol (2:1, v:v), followed by vigorous vortexing. Tubes were centrifuged for 5 min at 10,000 xg at room temperature to separate the organic bottom phase containing labeled SM and the aqueous upper phase containing ^14^C-labeled-phosphorylcholine. An aliquot (50 μl) of the upper phase was transferred to a vial with 10 ml of scintillant and counted for 2 min on a Beckman LS-6000IC scintillation counter. Enzyme activity was directly proportional to the disintegrations per min (dpm) resulting from the generation of ^14^C-phosphorylcholine. This assay was used to confirm the activity of hits and to determine IC_50_ values of confirmed compounds by evaluating the activity of human nSMase2 in duplicate at 8 concentrations of the compound of interest (concentration range 100 pM-1 mM).

### Library screening

The library of pharmacologically active compounds (LOPAC, Sigma) and a subset of the Spectrum Collection (Food and Drug Administration, MicroSource Discovery Systems) were screened against human nSMase2. This screening was done in 384-well format at a single concentration (10 μM) in duplicate using the fluorescence-based activity assay. Positive control inhibitors known to block nSMase2 activity were used in all assays: 150 μM GW4869 (Sigma), 100 μM manumycin A (Enzo Lifescience) and 100 μM altenusin (Enzo Lifescience). A negative control, 100 μM zoledronic acid (Enzo Lifescience), known to inhibit acid but not neutral sphingomyelinase, was also included in all plates. Compounds that showed at least 50% inhibitory activity selective for nSMase2 were considered hits. The compound physicochemical properties and drug-like features were used to evaluate the hits and select those to be tested in the confirmatory direct activity assay using ^14^C-labeled substrate.

### *In vitro* assessment of hippocampal neuronal survival and dendritic damage

All animal procedures were approved by the Johns Hopkins University Animal Care and Use Committee. Primary hippocampal neurons were prepared from day 18 decapitated embryos of Sprague-Dawley rats following previously described methods [15]. Cells were seeded on polyethyleneimine (PEI)-coated slides in 12-well plates and cultured between 14–21 days. Treatment was done with 100 ng/ml TNF-α or IL-1β in neurobasal medium without B27 supplement, in the presence of vehicle, cambinol (compound 1), an inactive cambinol analog (compound 2), zoledronic acid or SIRT1/2 inhibitors sirtinol and CHIC-35. After 18 h, cells were stained with 50 μg/ml Hoeschst 33342 for 20 min and then fixed with 4% paraformaldehyde for 30 min. The number of living and apoptotic cells was determined by fluorescence microscopy. A minimum of 500 cells were counted per treatment condition. Results were normalized to control untreated cells and were representative of at least two independent experiments conducted in triplicate. Statistical evaluation of the data was done by Student’s t-test. The *p* values <0.05 were considered statistically significant.

Quantification of neuronal morphology was done in primary hippocampal neurons plated in PEI-coated ultra-thin and optically clear flat bottom 96-well plates (Corning). After 14 days *in vitro*, neurons were treated with cambinol (0.1–30 μM) 15 min prior to exposure to TNF-α (100 ng/ml) for 18 h. Neurons were then fixed with 4% paraformaldehyde, and permeabilized with 0.1% Triton X-100 in phosphate-buffered saline (PBS-T) for 20 min at room temperature. Blocking was done for 1 h with 5% normal goat serum solution, then incubation with anti-MAP2 antibody (1:500, Sigma) was done, followed by three washes with PBS-T and 2 h incubation with Alexa 595 secondary antibody (1:500, Life Technologies). Dendrites were imaged with a 20X objective using a Zeiss Axio Observer Z1 microscope equipped with an Orca ER CCD camera (Hamamatsu Inc). A 6x6 image was obtained from each culture well, and a montage was created using Neurolucida imaging software (mbf Biosecience). The effects of the experimental treatments on dendrite length were determined and the results presented are the average of 5–6 individual experiments. Statistical significance was determined by one-way ANOVA analysis.

### Lipid extraction and ceramide measurements

Primary cortical neurons were prepared from embryonic day 18 Sprague-Dawley rats following previously described methods [15]. Cells were treated with 100 ng/mL TNF-α, 10 μM cambinol or both for 2 min. A crude lipid extract from cortical neurons was obtained using a modified Bligh and Dyer procedure [48]. Ceramide C12:0 (Avanti Polar Lipids) was included as an internal standard. The organic layers were dried in a nitrogen evaporator (Organomation Associates Inc.) and stored at -80°C. Dried organic layers were resuspended in methanol just prior to analysis. The detection and quantitation of individual ceramide species was performed on a high-performance liquid chromatography (HPLC) coupled electrospray ionization triple quadrupole mass spectrometer (API3000, AB Sciex Inc.) using settings similar to those described in previous studies [13]. Samples were handled by a CTC PAL autosampler (LEAP Technologies Inc.) and injected into an HPLC (PerkinElmer) equipped with a reverse phase C18 column (Phenomex). The column was first pre-equilibrated for 0.5 min with the first mobile phase consisting of 85% methanol, 15% H_2_O and 5 mM ammonium formate then eluted with the second mobile phase consisting of 99% methanol, 1% formic acid and 5 mM ammonium formate at the flow rate of 400.0 μl/min. The eluted sample was injected into the electrospray ionization source and detection of each ceramide species was conducted by multiple reaction monitoring. Spectral analysis was conducted using MultiQuant (AB Sciex Inc.), individually validated and normalized to the internal standard. Final data for each ceramide species in counts per second was expressed as the ratio of analyte/internal standard.

## Results

### Expression and activity of human nSMase2

**Fig 3:**
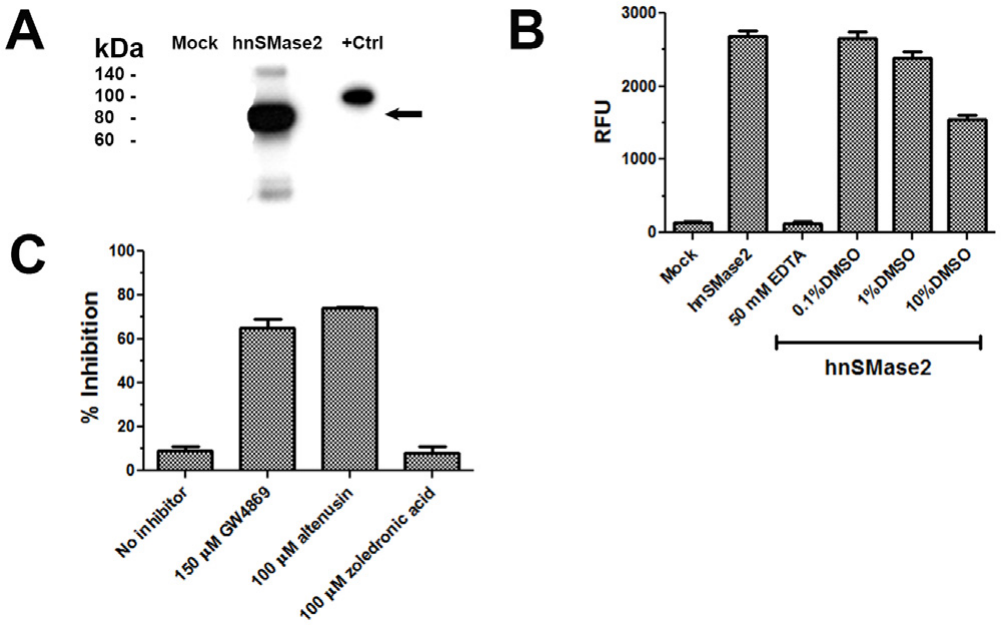
Expression and characterization of recombinant human nSMase2. **(A)** Western blot showing expression of recombinant human nSMase2-Flag tagged construct in stably transfected HEK293 cells. Positive control corresponds to GST-tagged human nSMase2 construct commercially available. **(B)** Human nSMase2 activity dependence with respect to the presence of EDTA and DMSO. **(C)** Modulation of enzymatic activity by known nSMase inhibitors (GW4869 and altenusin) and by a prototype aSMase specific inhibitor (zoledronic acid). Concentration of GW4869 is a nominal concentration based on dilutions from the stock solution; due to the low aqueous solubility of GW4869, the actual concentration is likely lower than 150 μM.

Full length recombinant human nSMase2 was constitutively expressed in stably transfected HEK293 cells. Fig 3A shows a Western blot with lysates of transfected HEK293 cells where a ~78 kDa protein is recognized by a polyclonal antibody against human nSMase2. Recombinant human nSMase2 displayed Mg^2+^-dependent SM-hydrolyzing activity and no significant inhibition of activity was seen with DMSO up to 1% (Fig 3B). Endogenous SM hydrolyzing activity of mock transfected HEK293 cells was negligible (not shown). Fig 3C shows that it was possible to inhibit enzyme activity with known nSMase inhibitors such as altenusin and GW4869, but not with the aSMase specific inhibitor, zoledronic acid. Enzyme stability and activity was preserved for up to six months when stored at -80°C.

### Characterization of nSMase2 activity assays

**Fig 4:**
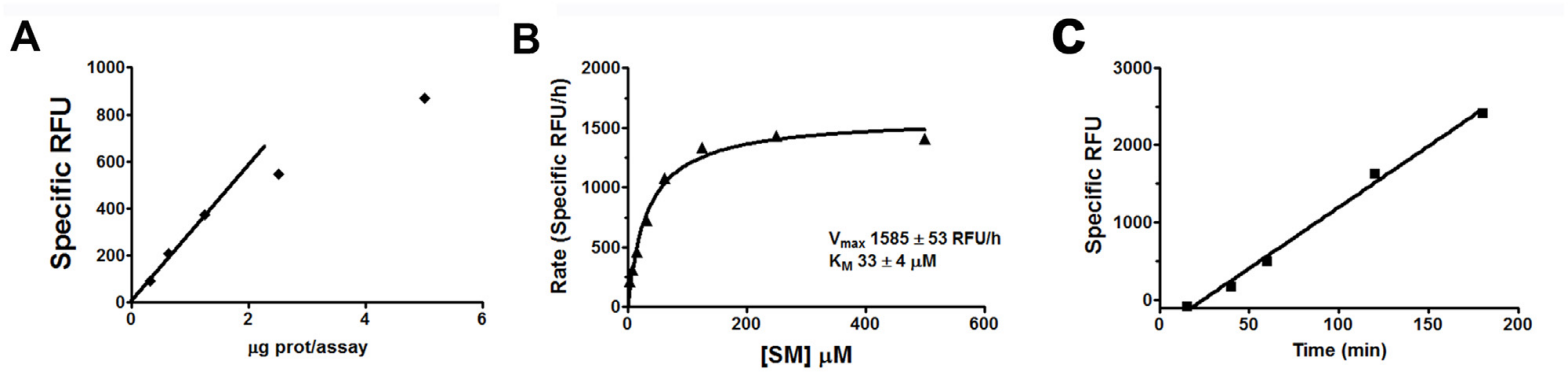
Characterization of fluorescence based activity assay for human nSMase2. Plots show the dependence of enzymatic activity with respect to **(A)** protein concentration in the presence of 250 μM SM for 1 h; **(B)** substrate concentration for 0.6 μg of protein from human nSMase2-containing cell lysate for 1 h; and **(C)** time in the presence of 0.6 μg of protein from nSMase2 expressing cell lysate and 250 μM SM. Data for activity *vs*. substrate concentration were fitted by non-linear least squares fitting to the Michaelis-Menten equation to determine *K*_m_ and *V*_max_.

Human nSMase2 activity assay based on monitoring resorufin fluorescence was characterized with respect to time, protein and substrate concentrations (Fig 4). We showed that optimal assay conditions (10–12 ng/μL nSMase2 lysate, 250 μM SM, 1 h at 37°C) in 384-well format had a signal to background ratio ≥ 3-fold and Z’ values were between 0.6 and 0.9.

The ^14^C-SM-labeled assay using human nSMase2 was also characterized with respect to time, protein and substrate concentrations (S1 Fig). Conditions utilized for the confirmatory screening were 0.5–1 μg nSMase2 lysate, 20 μM [N-methyl-^14^C] SM, for 1 h at 37°C.

### Library screening

**Fig 5:**
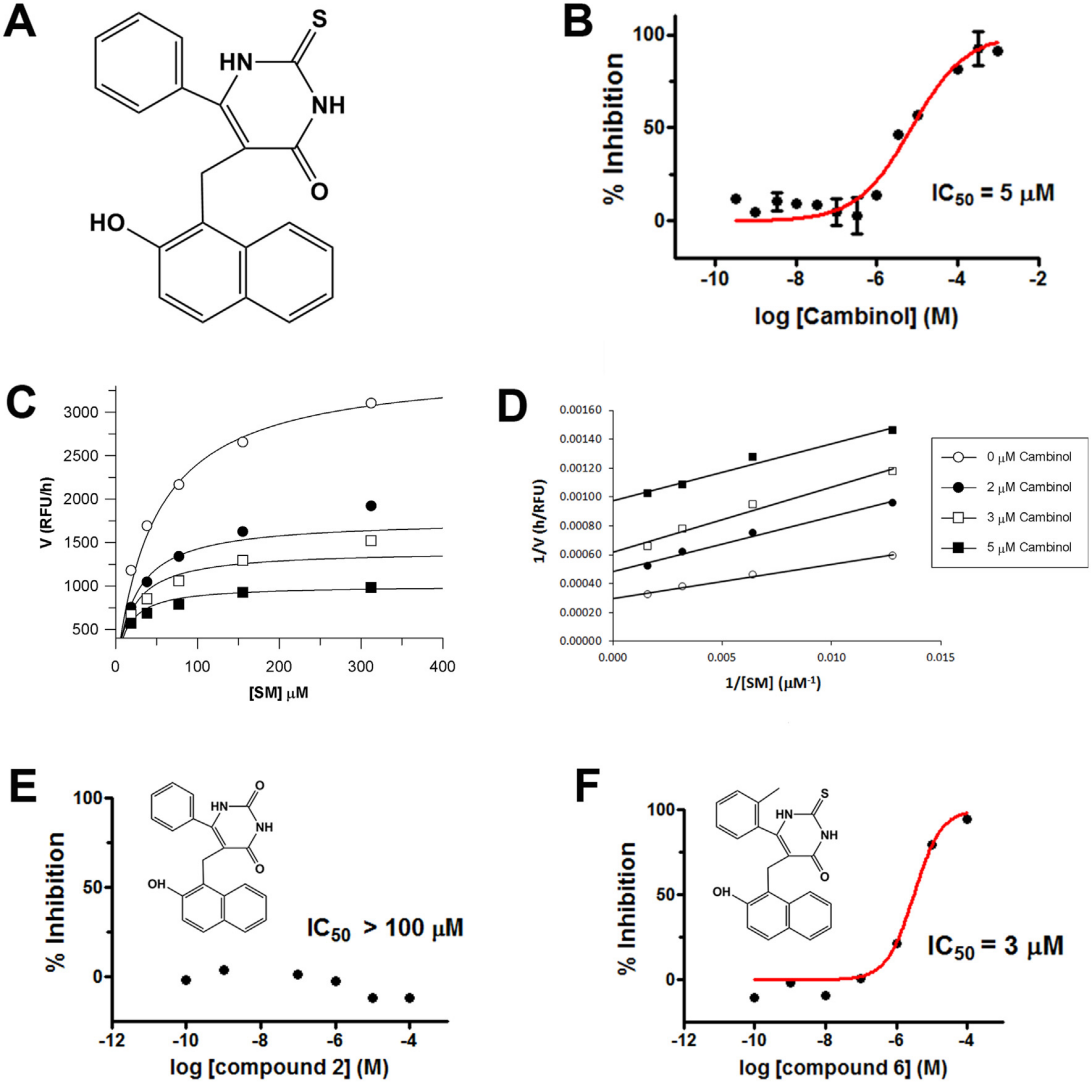
Inhibition of human nSMase2 by cambinol and closely related analogs. **(A)** Cambinol (compound 1) structure. **(B)** Dose response curve for inhibition of human nSMase2 by increasing concentrations of cambinol ([^14^C]-SM assay). **(C)** Non-linear least squares fit of the Michaelis-Menten equation for the activity of human nSMase2 *vs*. SM concentration in the presence of different concentrations of cambinol. *K*_m_ and *V*_max_ values decrease with increasing concentrations of inhibitor. **(D)** Lineweaver-Burk plot for data shown in (C). **(E)** Dose response curve for inhibition of human nSMase2 by cambinol analog compound 2. **(F)** Dose response curve for inhibition of human nSMase2 by cambinol analog compound 6.

**Fig 6:**
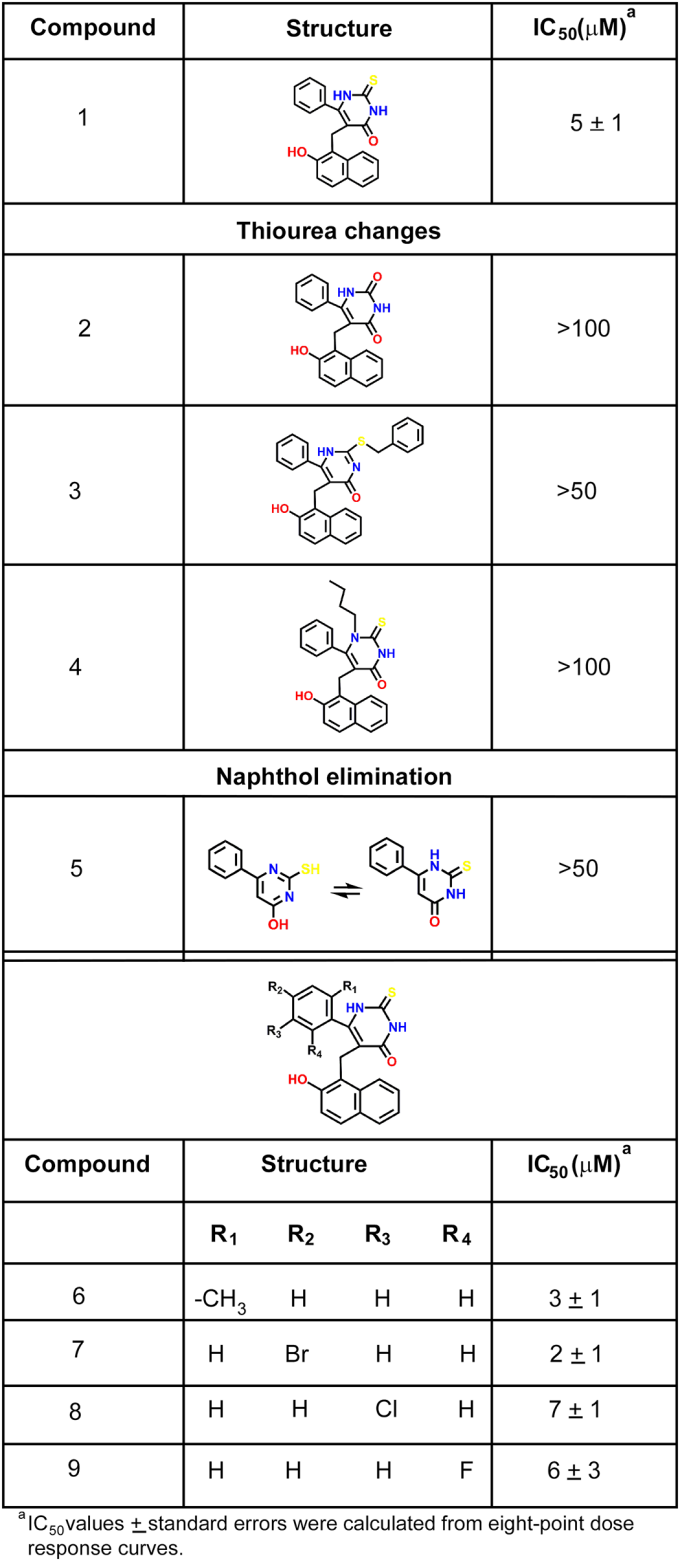
Structure-activity relationship study for cambinol and human nSMase2. Table illustrating the IC_50_ values for various analogs of cambinol in the direct radioactive assay using ^14^C-labeled substrate.

A total of 2320 compounds from the LOPAC and FDA libraries were tested in 384-well format in duplicate at a single concentration (10 μM). Seventy three compounds showed at least 50% inhibitory activity and were considered active. Based on the Lipinski’s rule of 5 [49] and other criteria such as presence of moieties known to undergo metabolic reactions, 23 drug-like compounds were chosen from the list of hits. This latter group was then tested in the counter screening assay and the radioactive confirmatory assay and only one compound, cambinol (Fig 5A and Fig 6, compound 1), showed no interference with the fluorescence assay and >80% inhibition at 10 μM in the direct human nSMase2 activity assay. An IC_50_ value of 5 ± 1 μM was determined for cambinol using the confirmatory direct activity assay (Fig 5B).

### Cambinol is an uncompetitive inhibitor for human nSMase2

Dependence of the rate of SM hydrolysis on substrate concentration in the presence of different concentrations of cambinol showed that increasing concentrations of cambinol caused a decrease of *K*_m_ and *V*_max_ (Fig 5C). This is the hallmark of uncompetitive inhibition. Moreover, the corresponding Lineweaver-Burk plot exhibits parallel lines (Fig 5D) characteristic of this mode of inhibition. Non-linear least squares fit of the data (Fig 5C) showed that the affinity of cambinol for human nSMase2 (*K*_i_) was 7 μM.

Due to cambinol’s uncompetitive mode of inhibition and the presence of the potentially reactive thiourea in its structure, we assessed the time-dependence of its inhibition. S1 Table shows that IC_50_ values for cambinol did not change with incubation time.

### Preliminary Structure Activity Relationship (SAR) Studies

Prior reports suggest that cambinol is also an inhibitor of SIRT1 and SIRT2 [50]. The relationship between structure and activity for cambinol was studied with 8 analogs (Fig 6) originally synthesized to examine cambinol’s SAR against SIRT1 and SIRT2 [51,52]. Fig 5E and 5F show the dose response curves obtained for compounds 2 and 6, as examples of an inactive and an active analog, respectively. We found that the thiourea portion of the molecule was essential for activity as evidenced by the loss of activity on substitution of the S atom with O (compound 2), addition of a benzyl group to the S atom (compound 3) or addition of an N-*n*-butyl group (compound 4). Moreover, the naphthol portion of the molecule also appears to be required for activity as suggested by the 10 fold increase in IC_50_ value for compound 5. Finally, substitutions on the benzene ring do not appear to have a significant effect on compound inhibitory activity (compounds 6–9). Interestingly, it was not possible to block human nSMase2 activity using SIRT1/2 inhibitors sirtinol or CHIC-35 even at 100 μM (S2 Fig). Sirtinol has an IC_50_ of 131 μM for SIRT1 and 38 μM for SIRT2 [53,54], while CHIC-35 has an IC_50_ of 98 nM for SIRT1 and 19.6 μM for SIRT2 [55]. These results indicate that cambinol’s nSMase2 inhibitory activity is not shared by other SIRT1/2 prototype inhibitors.

### Inhibition of rat nSMase2 in primary neurons protects against cytokine-induced ceramide production, cell death and dendritic damage

**Fig 7:**
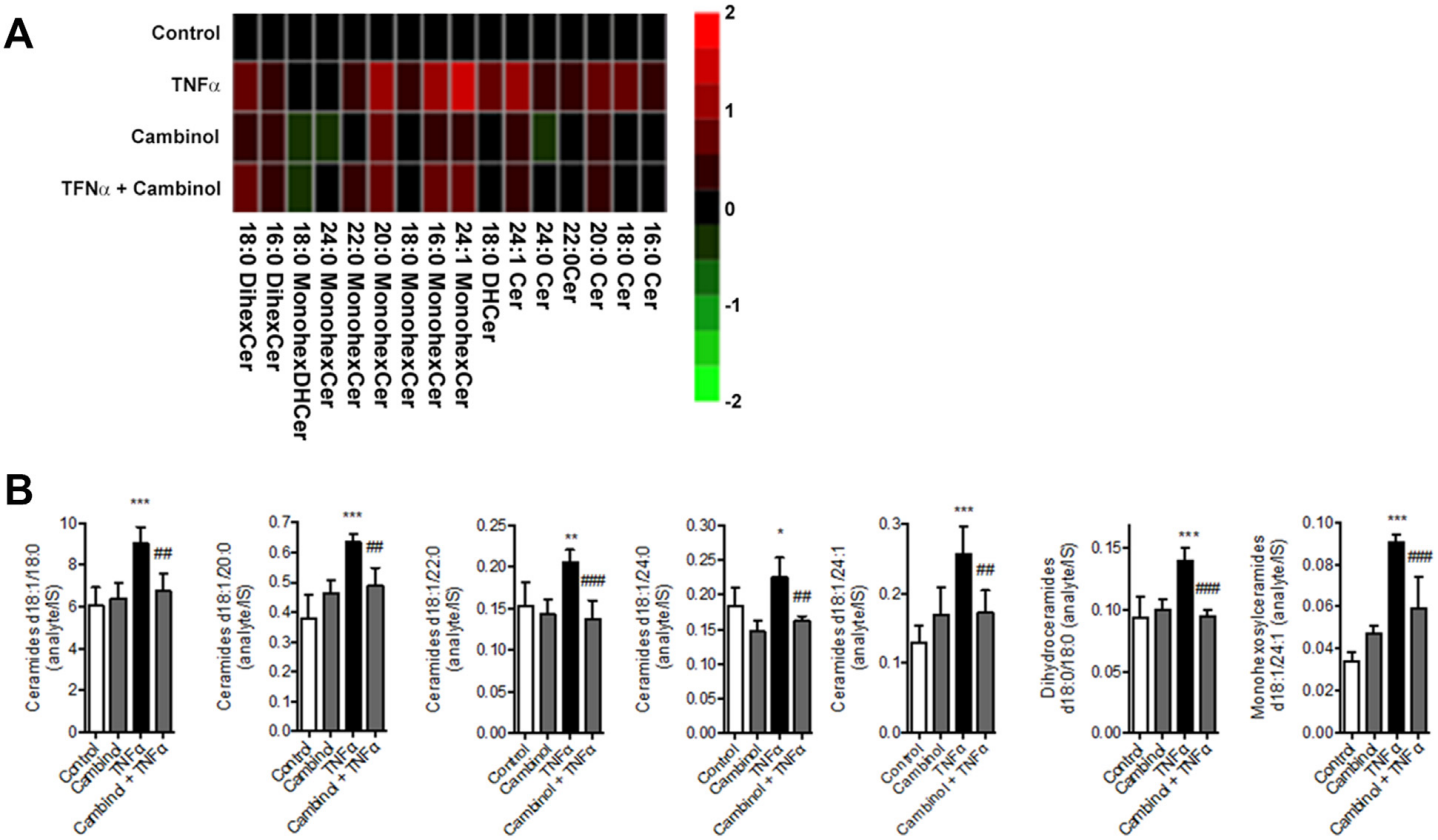
Effect of cambinol on TNF-α-induced changes in ceramide profiles of rat primary neurons. **(A)** Heat-map represents ceramide profiles of rat primary neurons after treatment with TNF-α, cambinol or cambinol + TNF-α. Columns represent control, 100 ng/ml TNF-α for 2 min, 10 μM cambinol and cambinol + TNF-α. Drug was added to cells 15 min prior to TNF-α stimulation. Heat maps were generated based on normalization of the ceramide values using z transformations. Color scale illustrates quantitation, red color indicates increase of ceramide abundance and green color indicates depletion with respect to control treatment. **(B)** Quantitative analysis representation of ceramide levels for the four treatments shown in (A).

**Fig 8:**
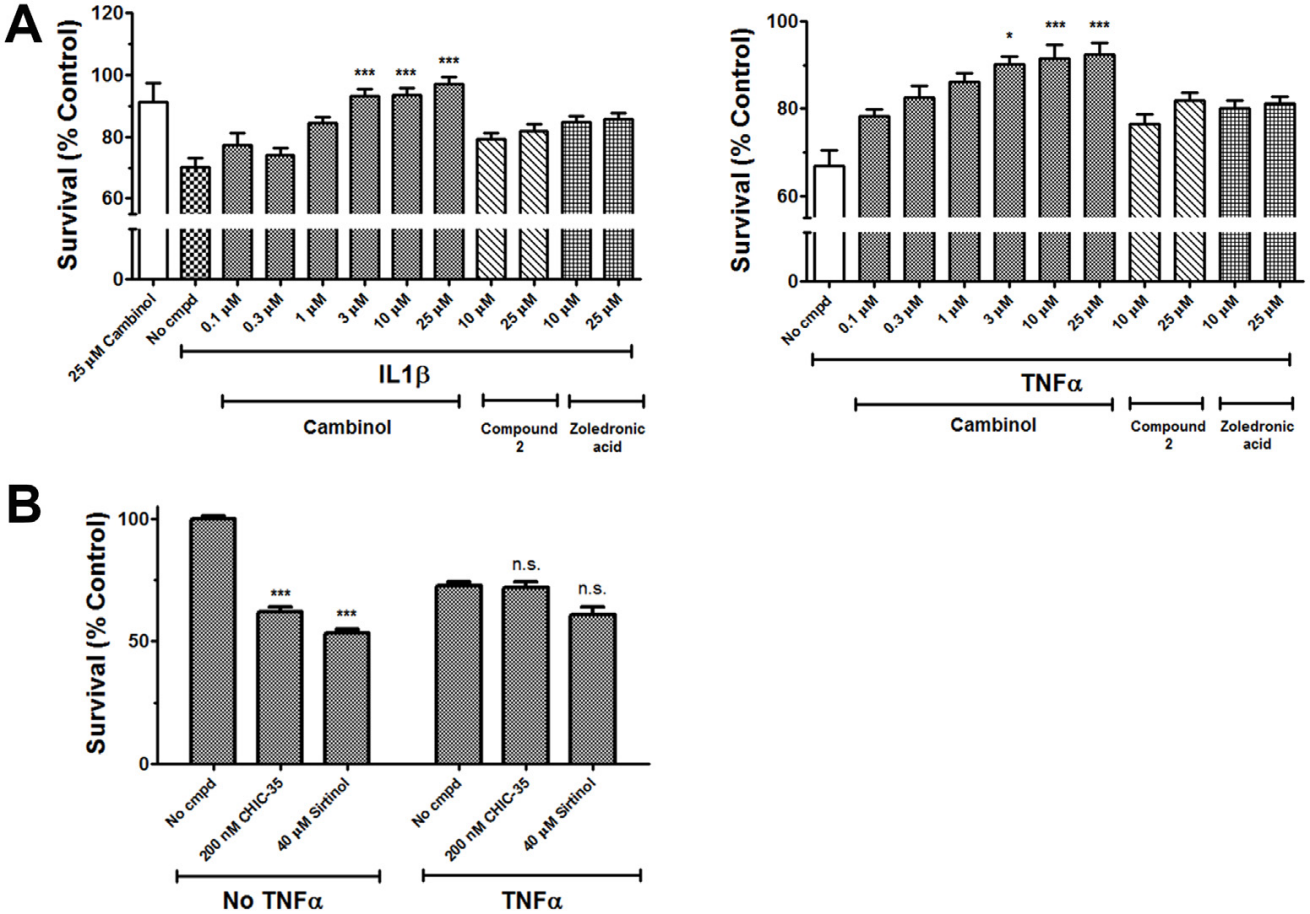
Effect of cambinol on TNF-α or IL-1β-induced loss of viability in neurons. **(A)** Effects of cambinol (0.1–25 μM), inactive analog compound 2 (10–25 μM) or zoledronic acid (10–25 μM) on the survival of rat primary neurons treated with TNF-α or IL-1β. (100 ng/ml for 18 h) **(B)** Effects of SIRT1/2 inhibitors CHIC-35 (200 nM) and sirtinol (40 μM) on TNF-α induced neuronal cell death. Compounds were added 15 min prior to cytokine. Error bars correspond to S.E.M. of at least 3 independent determinations. *** p < 0.001.

**Fig 9:**
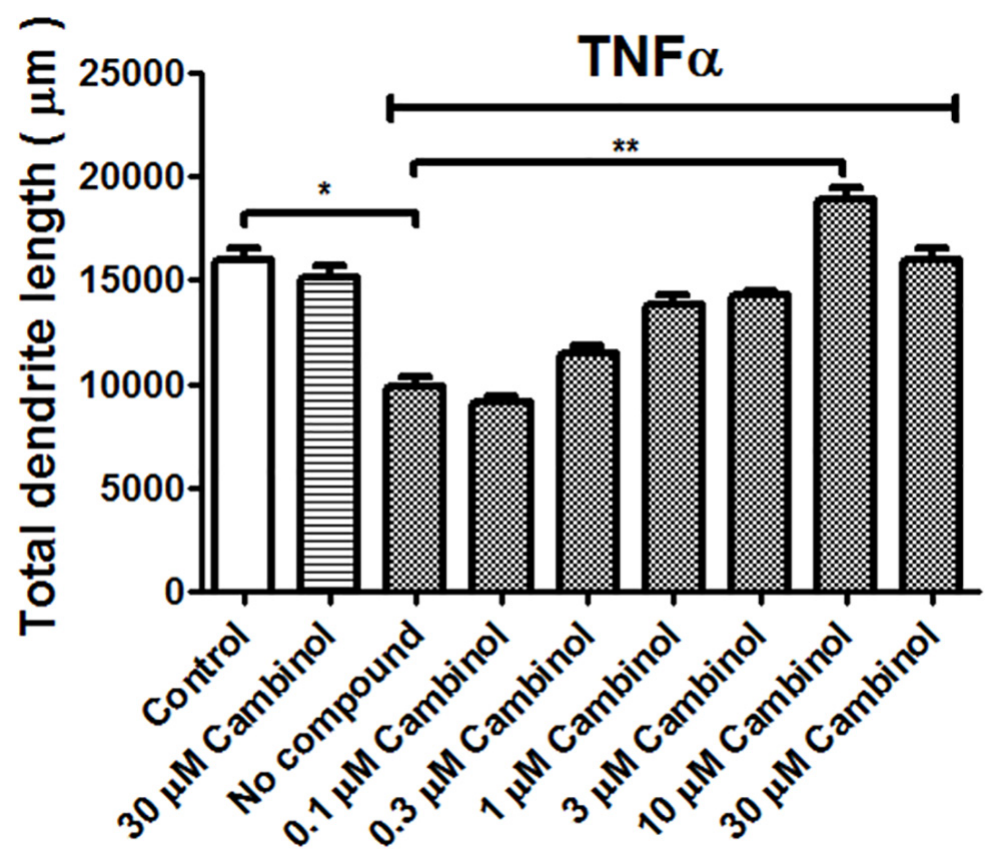
Effect of cambinol on TNF-α-induced decrease in dendrite length of rat primary hippocampal neurons. Cells were treated with cambinol (0.1–30 μM) and TNF-α (100 ng/ml) for 18 h. Dendrite length was assessed by imaging of MAP-2 stained cells using Neurolucida software. Results shown are the average of 5–6 experiments. Error bars correspond to S.E.M. * p < 0.05 and ** p < 0.01.

We also examined cambinol’s inhibitory activity using rat nSMase2 from brain homogenates and found it was as equally effective blocking SM hydrolysis (IC_50_ = 6 μM, S3 Fig). Treatment of rat neurons with IL-1β or TNF-α causes activation of nSMase2, which increases ceramide production and induces loss of cell viability [7,9,10]. Fig 7A and 7B show a mass spectrometric analysis of the lipid levels in rat primary cortical neurons. TNF-α induces a rapid increase of long chain ceramide levels that was blocked by cambinol. Moreover, Fig 8 shows that TNF-α or IL-1β-induced hippocampal neuron cell death, as assessed by Hoeschst 33342 staining was also inhibited by cambinol in a dose dependent manner, but not by the inactive analog, compound 2, the aSMase inhibitor zoledronic acid (Fig 8A) or the SIRT1/2 inhibitors, sirtinol, and CHIC-35 (Fig 8B). Similarly knocking down nSMase2 with a lentivirus shRNA construct protected neurons from TNFα-induced cell death indicating that both nSMase2 activity inhibition and decrease protein expression are associated with survival to pro-inflammatory stimuli in hippocampal neurons (S4 Fig). Finally, cambinol, in a dose-dependent manner, prevented TNFα-induced hippocampal neuron dendrite damage, assessed as decrease in dendritic length (Fig 9).

## Discussion

We characterized the activity of human nSMase2 using two enzymatic assays previously employed for the determination of bacterial, rat or bovine nSMase activity [43–45,56]. These assays were used to carry out the screening of two libraries of pharmacologically active compounds allowing the identification of cambinol as an uncompetitive inhibitor for human nSMase2. This compound was found to be capable of blocking nSMase2 activity *in vitro*, inhibiting pro-inflammatory cytokine-induced increases in ceramide and providing neuroprotection.

Discovery efforts for pharmacological inhibitors of nSMase activity have largely been focused on enzymes of bacterial, bovine and rat origin. Characterization of the bacterial enzymes has shed light on the tertiary structure and enzymatic mechanism of these proteins. It has been assumed that human nSMase2 is homologous to the bacterial enzyme based on the conservation of the residues involved in catalysis and metal ion chelation. However, the degree of conservation for the rest of the primary structure is rather low (approximately 20% sequence identity between human and *Bacillus cereus* proteins) [5].

We report that similar to the bacterial and rodent enzymes, recombinant human nSMase2 exhibited Mg^2+^-dependence and inhibition by GW4869, manumycin and altenusin, while not being affected by the aSMase specific inhibitor, zoledronic acid. In contrast to the rodent enzyme, presence of anionic phospholipids such as phosphatidylserine (PS) [4,47] did not significantly affect the human enzyme activity (S5 Fig). One possible reason for the marginal effect of PS on human nSMase2 activity could be due to the cell lysate preparation. Under these conditions the enzyme would still be interacting with endogenous lipids that are required for optimal activity. Although the fluorescence and the ^14^C-SM-based nSMase2 assays have been previously described, a systematic characterization using the human enzyme has not been published. We characterized both assays with respect to time, concentration of substrate and enzyme in order to determine the experimental conditions to carry a screening campaign which identified cambinol as a new human nSMase2 inhibitor.

Cambinol provides an alternative to the commonly used nSMase inhibitors depicted in Fig 2. When compared to GW4869, the most extensively used prototype, cambinol has similar potency but exhibits significantly higher aqueous solubility and lower molecular weight (MW). When compared to inhibitors with similar MW (e.g. altenusin, C11AG or macquarimicin A), it is a more potent inhibitor.

Cambinol was found to be a novel uncompetitive inhibitor of human nSMase2 suggesting that it binds to the enzyme-substrate complex. This is the first reported example of an uncompetitive inhibitor for human nSMase2. Given the presence of a thiourea moiety in cambinol’s structure, this compound could be acting as a time-dependent irreversible inhibitor. Consequently, we evaluated the effects of increasing cambinol-enzyme pre-incubation time on the inhibitory activity of the compound. We report that cambinol’s inhibition was independent of pre-incubation time up to 2 h. Cambinol’s mode of inhibition and the lack of time-dependence of its IC_50_ value indicate that cambinol does not bind to the substrate binding site of the enzyme but rather to an alternative site blocking activity and it does so reversibly. A search of the PubChem compound database indicates that cambinol is not a promiscuous compound based on its low hit rate (<10%) http://pubchem.ncbi.nlm.nih.gov/summary/summary.cgi?cid=3246390. Out of 245 biochemical and cell-based assays reported in the database for which cambinol has been tested, only 28 showed activity for the compound. From these, 13 were assays specific to probe SIRT1/2 activity or function and the rest included targets such as p450-CYP1a2, thyroid stimulating hormone receptor, and p53 expression. The findings that inhibition could be confirmed with independent readouts, that inhibition was inhibitor-enzyme incubation time independent and that cambinol exhibits a low promiscuity score indicate that this compound is a bona fide inhibitor of nSMase2 rather than a promiscuous inhibitor.

Despite the low amino acid sequence identity between mammalian and bacterial nSMases, in addition to inhibiting the human enzyme, cambinol was also found to inhibit *B*. *cereus* nSMase (not shown) and rat nSMase2 (S3 Fig) with IC_50_ = 5 and 6 μM, respectively. Inhibition of bacterial, rat and human enzymes suggests that binding of cambinol must occur to a conserved region of these proteins. The results indicate that cambinol could be used as a tool to study the activity of nSMase2 in murine animal models. From a selectivity standpoint, cambinol did not inhibit aSMase (IC_50_ > 10 μM), (personal communication with Drs. Marc Ferrer and Wei Zheng at the National Center for Advancing Translational Sciences, NCATS). Cambinol’s activity against nSMase1 and nSMase3 has not yet been determined. In future studies, it would also be interesting to assess if cambinol’s prevention of ceramide accumulation would trigger compensatory mechanisms in enzymes/transporters associated with ceramide processes.

Cambinol has previously been reported as an inhibitor of the NAD-dependent deacetylase activity of sirtuin, human silent information regulator type 1/2 (SIRT1/2), with IC_50_ values of 56 μM and 59 μM for SIRT1 and SIRT2, respectively [52]. Additionally, cambinol induced apoptosis in BCL6-expressing Burkitt lymphoma cell lines possibly due to BCL6 and p53 hyperacetylation consequence of SIRT1/2 inhibition [50]. Our results show that cambinol is approximately a 10-fold more potent human nSMase2 inhibitor compared to SIRT1/2.

Lugrin *et al*. [57] showed that cambinol, but not other SIRT1/2 inhibitors, effectively inhibited TNF-α and IL-6 release by macrophages prompting the authors to suggest that cambinol may have activity beyond SIRT1/2. It is possible that these effects could be, at least in part, due to the inhibition of nSMase2. To further study this, we evaluated the effects of cambinol and nSMase-specific shRNA in rat primary hippocampal neurons. We found that cambinol decreased ceramide levels and protected neurons from TNF-α or IL-1β-induced cell death and TNF-α-induced dendritic damage. The involvement of nSMase2 inhibition in these protective effects was supported by the use of shRNA targeting nSMase2 expression which also provided protection against apoptosis.

Tests of the inhibitory activities of 8 cambinol analogs allowed the identification of the thiourea and to a lesser degree the naphthol portions of cambinol as essential moieties for activity. Interestingly, the addition of an *n*-butyl to the N atom of the thiourea (compound 4) eliminated its human nSMase2 activity, while Medda *et al*. reported that this change significantly improved the potency and specificity against SIRT 2 [52]. These findings suggest that the pharmacophore for SIRT1/2 inhibition differs from the one for human nSMase2 inhibition possibly allowing the separation of the two activities by further structural modifications of cambinol. Additional structural substitutions are currently being considered to improve the potency and selectivity of cambinol.

It is important to point out that some thiourea containing compounds have been reported to be chemically reactive and/or goitrogenic, while some others can cause hypersensitivity reactions and are pulmonary or hepatic toxins [58]. On the other hand cambinol has been shown to be effective and well tolerated as an anti-cancer agent in a Burkitt lymphoma xenografts model in mice, even at doses as high as 100 mg/kg intravenously [50]. We are currently carrying additional SAR and specificity studies in an effort to find cambinol analogues that are potent and specific inhibitors of nSMase2. In summary, modulation of human nSMase2 activity could be a therapeutic approach worth pursuing for the treatment of a number of neurodegenerative diseases and cambinol could be used as an alternative prototype inhibitor with similar inhibitory potency but improved solubility over currently available compounds.

## Supporting Information

S1 Fig Characterization of radioactivity-based assay for human nSMase2. Plots show the linear ranges for enzymatic activity with respect to **(A)** protein concentration, **(B)** substrate concentration and **(C)** time. Data for activity *vs*. substrate concentration were fitted by non-linear least squares fitting to the Michaelis-Menten equation to determine *Km* and *V*_max_.
(DOCX)

S2 Fig Effect of SIRT1/2 inhibitors on the activity of human nSMase2. Dose response curves for sirtinol and CHIC-35 evaluated in the fluorescence nSMase2 assay showing their lack of effects on human nSMase2 activity (IC_50_ > 100 μM and 70 μM, respectively). In comparison, sirtinol has an IC_50_ = 131 μM for SIRT1 and 38 μM for SIRT2, and CHIC-35 IC_50_ = 98 nM for SIRT1 and 19.6 μM for SIRT2 [references in S1 Supporting Information]. Results are the average of two independent experiments.
(DOCX)

S3 Fig Inhibition of rat nSMase2 activity by cambinol. A membrane protein-enriched fraction of rat brain homogenate was used as the source for nSMase2. Test of cambinol inhibitory activity was performed following the same optimal conditions used for the human enzyme in the fluorescence assay. Results are the average of two independent experiments.
(DOCX)

S4 Fig Effects of nSMase2 knock down on hippocampal neurons TNFα-induced apoptosis. Lentivirus packed with piLenti-siRNA-GFP specific against rat nSMase2 was used to transduce rat primary hippocampal neurons (MOI 5, Abm Inc). Cells were treated with 100 ng/ml TNF-α for 18 h and apoptotic nuclei were assessed as specified in the manuscript by staining cells with Hoechst 33342. Sc: scrambled siRNA, used as negative control.
(DOCX)

S5 Fig Effects of PS on the activity of human nSMase2 monitored in the fluorescence assay. Enzyme substrate was either 20 μM SM or 20 μM SM/PS at equimolar concentration in assay buffer containing 0.1% TX-100. Results are the average of two independent experiments.
(DOCX)

S1 Supporting Information Supporting information figure legends, references and raw data for Figs 1–7. (DOCX)

S1 Table Time dependence study of cambinol inhibitory activity against human hSMase2. (DOCX)
