# Supplementary figures and images for: Cambinol, a Novel Inhibitor of Neutral Sphingomyelinase 2 Shows Neuroprotective Properties
Source: PLoS One. 2015 May 26;10(5):e0124481. doi: 10.1371/journal.pone.0124481 (PMC4444023; doi:10.1371/journal.pone.0124481)

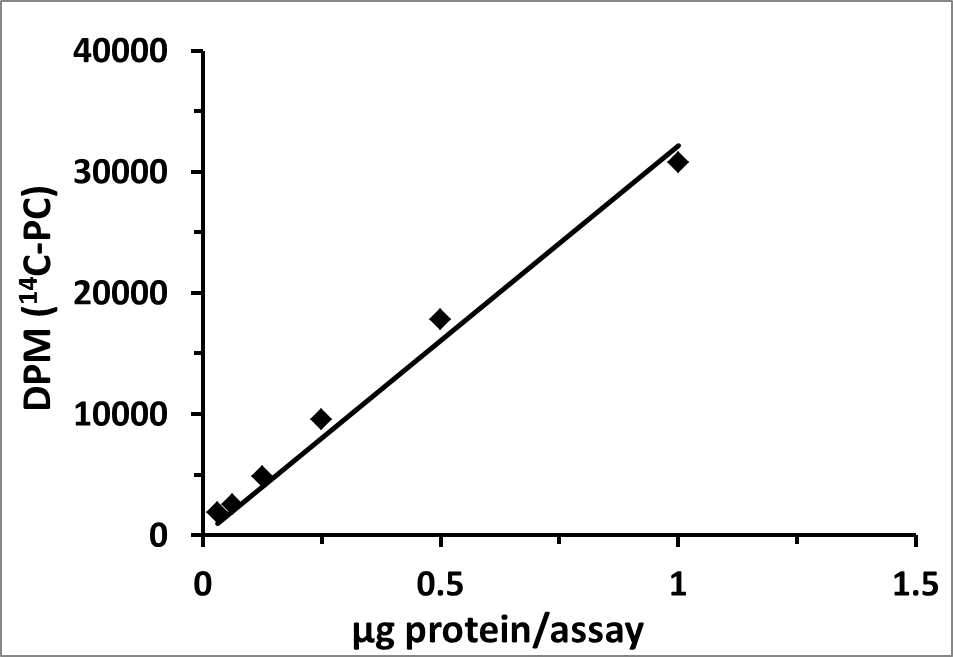
**Figure S1**

**(A)**


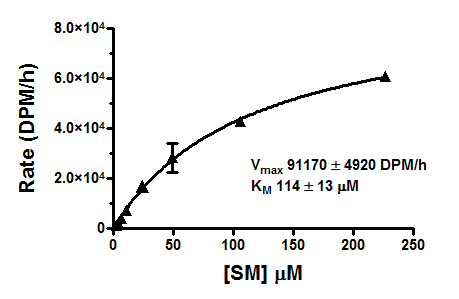


**(B)**

**
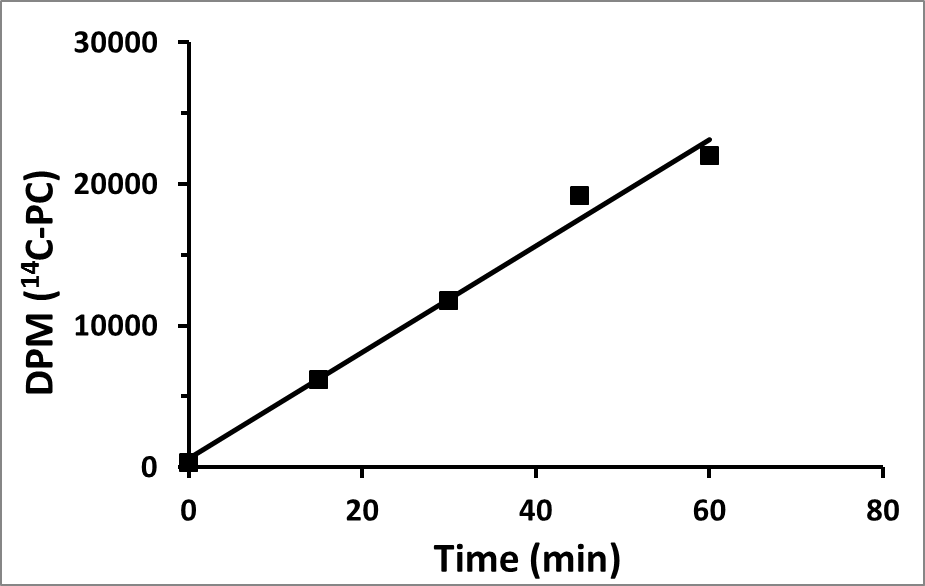
(C)**

Supplement: S1 Fig — Plots show the linear ranges for enzymatic activity with respect to (A) protein concentration, (B) substrate concentration and (C) time. Data for activity vs. substrate concentration were fitted by non-linear least squares fitting to the Michaelis-Menten equation to determine Km and V max. (DOCX) [file pone.0124481.s001.docx]

**Figure S2**


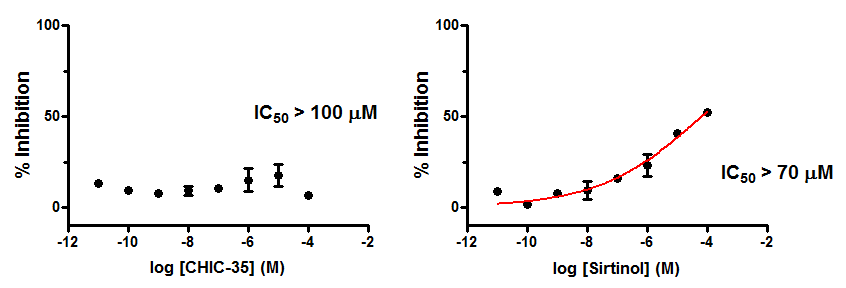

Supplement: S2 Fig — Dose response curves for sirtinol and CHIC-35 evaluated in the fluorescence nSMase2 assay showing their lack of effects on human nSMase2 activity (IC50 > 100 μM and 70 μM, respectively). In comparison, sirtinol has an IC50 = 131 μM for SIRT1 and 38 μM for SIRT2, and CHIC-35 IC50 = 98 nM for SIRT1 and 19.6 μM for SIRT2 [references in S1 Supporting Information]. Results are the average of two independent experiments. (DOCX) [file pone.0124481.s002.docx]

**Figure S3**

**
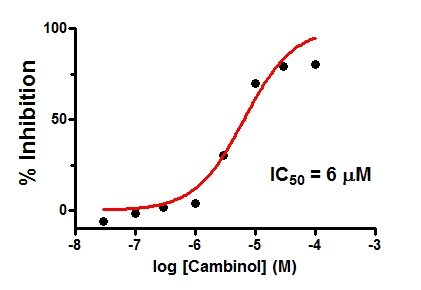
**

Supplement: S3 Fig — A membrane protein-enriched fraction of rat brain homogenate was used as the source for nSMase2. Test of cambinol inhibitory activity was performed following the same optimal conditions used for the human enzyme in the fluorescence assay. Results are the average of two independent experiments. (DOCX) [file pone.0124481.s003.docx]

**Figure S4**

**
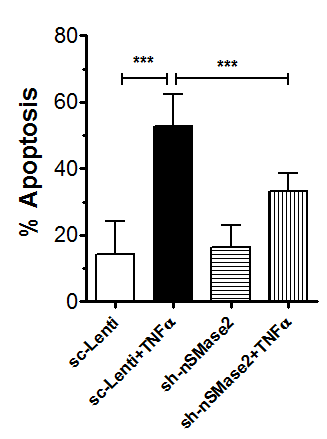
**

Supplement: S4 Fig — Lentivirus packed with piLenti-siRNA-GFP specific against rat nSMase2 was used to transduce rat primary hippocampal neurons (MOI 5, Abm Inc). Cells were treated with 100 ng/ml TNF-α for 18 h and apoptotic nuclei were assessed as specified in the manuscript by staining cells with Hoechst 33342. Sc: scrambled siRNA, used as negative control. (DOCX) [file pone.0124481.s004.docx]

**Figure S5**

**
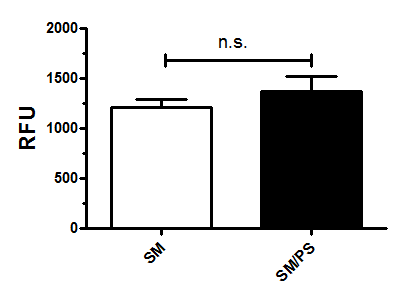
**

Supplement: S5 Fig — Enzyme substrate was either 20 μM SM or 20 μM SM/PS at equimolar concentration in assay buffer containing 0.1% TX-100. Results are the average of two independent experiments. (DOCX) [file pone.0124481.s005.docx]
