# Supplementary material for: Cambinol, a Novel Inhibitor of Neutral Sphingomyelinase 2 Shows Neuroprotective Properties
Source: PLoS One. 2015 May 26;10(5):e0124481. doi: 10.1371/journal.pone.0124481 (PMC4444023; doi:10.1371/journal.pone.0124481)
Supplement: S1 Supporting Information — (DOCX) [file pone.0124481.s006.docx]

# Supporting Information Figure Legends

**Figure S1. Characterization of** r**adioactivity-based assay for human nSMase2.** Plots show the linear ranges for enzymatic activity with respect to **(A)** protein concentration, **(B)** substrate concentration and **(C)** time. Data for activity vs. substrate concentration were fitted by non-linear least squares fitting to the Michaelis-Menten equation to determine *K*_m_ and *V*_max_.

**Figure S2. Effect of SIRT1/2 inhibitors on the activity of human nSMase2.** Dose response curves for sirtinol and CHIC-35 evaluated in the fluorescence nSMase2 assay showing that they are not effective inhibitors of human nSMase2 (IC50 > 100 μM and 70 μM, respectively). In comparison, sirtinol has an IC_50_ = 131 μM for SIRT1 and 38 μM for SIRT2 [[1](#_ENREF_1),[2](#_ENREF_2)], and CHIC-35 has an IC_50_ = 98 nM for SIRT1 and 19.6 μM for SIRT2 [[3](#_ENREF_3)]. Inhibitors were evaluated in the fluorescence nSMase2 assay. Results are the average of two independent experiments.

**Figure S3. Inhibition of rat nSMase2 activity by cambinol.** A membrane protein-enriched fraction of rat brain homogenate was used as the source for nSMase2. Test of cambinol inhibitory activity was performed following the same optimal conditions used for the human enzyme in the fluorescence assay. Results are the average of two independent experiments.

**Figure S4. Effects of nSMase2 knock down on hippocampal neurons TNFα-induced apoptosis.** Lentivirus packed with piLenti-siRNA-GFP specific against rat nSMase2 was used to transduce rat primary hippocampal neurons (MOI 5, Abm Inc). Cells were treated with 100 ng/ml TNF-α for 18 h and apoptotic nuclei were assessed as specified in the manuscript by staining cells with Hoechst 33342. Sc: scrambled siRNA, used as negative control.

**Figure S5. Effects of phosphatidylserine (PS) on the activity of human nSMase2 monitored in the fluorescence assay.** Enzyme substrate was either 20 μM SM or 20 μM SM/PS at equimolar concentration in assay buffer containing 0.1% TX-100. Results are the average of two independent experiments.

## References

1. Grozinger CM, Chao ED, Blackwell HE, Moazed D, Schreiber SL (2001) Identification of a class of small molecule inhibitors of the sirtuin family of NAD-dependent deacetylases by phenotypic screening. J Biol Chem 276: 38837-38843.

2. Mai A, Massa S, Lavu S, Pezzi R, Simeoni S, et al. (2005) Design, synthesis, and biological evaluation of sirtinol analogues as class III histone/protein deacetylase (Sirtuin) inhibitors. J Med Chem 48: 7789-7795.

3. Napper AD, Hixon J, McDonagh T, Keavey K, Pons JF, et al. (2005) Discovery of indoles as potent and selective inhibitors of the deacetylase SIRT1. J Med Chem 48: 8045-8054.

## Raw data

**Figure 2B**

| Treatment | RFU | | | |
| --- | --- | --- | --- | --- |
| Mock | 118.8493 | 104.9372 | 171.2852 |  |
| hnSMase2 | 2847.5130 | 2802.3110 | 2597.3610 | 2477.3270 |
| 50 mM EDTA | 148.5635 | 175.3315 | 119.4525 | 64.0735 |
| 0.1%DMSO | 2703.2500 | 2871.2510 | 2454.0980 | 2595.8260 |
| 1%DMSO | 2256.2870 | 2299.5150 | 2360.8330 | 2629.8220 |
| 10%DMSO | 1706.1510 | 1610.6720 | 1424.4530 | 1442.3390 |

**Figure 2C**

| Treatment | RFU | | | |
| --- | --- | --- | --- | --- |
| No inhibitor | 6.6 | 11.2 |  |  |
| 150 μM GW4869 | 59.6 | 72.8 | 57.5 | 70.4 |
| 100 μM altenusin | 75.4 | 73.8 | 72.1 | 74.7 |
| 100 μM zoledronic acid | 5.2 | 10.9 |  |  |

**Figure 3A**

| Protein (μg) | RFU |
| --- | --- |
| 0.3125 | 93 |
| 0.6250 | 208 |
| 1.2500 | 373 |
| 2.5000 | 546 |
| 5.0000 | 869 |

**Figure 3B**

| [SM] (μM) | RFU |
| --- | --- |
| 500.00 | 1408.02 |
| 250.00 | 1431.87 |
| 125.00 | 1331.12 |
| 62.50 | 1075.56 |
| 31.25 | 722.04 |
| 15.63 | 459.12 |
| 7.81 | 311.45 |
| 3.91 | 212.26 |

**Figure 3C**

| Time (min) | RFU |
| --- | --- |
| 15 | -76.07 |
| 40 | 176.04 |
| 60 | 506.20 |
| 120 | 1636.91 |
| 180 | 2410.94 |

**Figure 4B**

| Log [Cambinol] | %Inhibition | |
| --- | --- | --- |
| -3.00000 | 92.0 | 91.0 |
| -3.47756 | 99.0 | 86.0 |
| -4.00000 | 83.0 | 80.0 |
| -5.00000 | 56.0 | 57.0 |
| -5.47756 | 46.0 | 47.0 |
| -6.00000 | 13.0 | 15.0 |
| -6.47756 | -4.0 | 10.0 |
| -7.00000 | 0.0 | 10.0 |
| -7.47756 | 8.0 | 10.0 |
| -8.00000 | 8.0 | 11.0 |
| -8.47756 | 14.0 | 7.0 |
| -9.00000 | 6.0 | 4.0 |
| -9.47756 | 12.0 | *18.0** |

**Figure 4C**

|  | V (RFU/h) | | | |
| --- | --- | --- | --- | --- |
| [SM] (μM) | 0 μM Cambinol | 2 μM Cambinol | 3 μM Cambinol | 5 μM Cambinol |
| 19.5 | 912 | 641 | 571 | 500 |
| 39.0 | 1175 | 752 | 663 | 567 |
| 78.0 | 1688 | 1042 | 849 | 684 |
| 156.0 | 2161 | 1336 | 1055 | 784 |
| 313.0 | 2651 | 1621 | 1288 | 922 |

**Figure 4D**

|  | 1/V (h/RFU) | | | |
| --- | --- | --- | --- | --- |
| 1/[SM] (μM^-1^) | 0 μM Cambinol | 2 μM Cambinol | 3 μM Cambinol | 5 μM Cambinol |
| 0.013 | 0.00059 | 0.00096 | 0.00118 | 0.00146 |
| 0.006 | 0.00046 | 0.00075 | 0.00095 | 0.00128 |
| 0.003 | 0.00038 | 0.00062 | 0.00078 | 0.00109 |
| 0.002 | 0.00032 | 0.00052 | 0.00066 | 0.00102 |

**Figure 4E**

| Log [Compound 2] | % Inhibition | |
| --- | --- | --- |
| -4.0 | -11.0 | -13.0 |
| -5.0 | -11.0 | -12.0 |
| -6.0 | -2.0 | -3.0 |
| -7.0 | 3.0 | 0.0 |
| -9.0 | 4.0 | 4.0 |
| -10.0 | -1.0 | -2.0 |

**Figure 4F**

| Log [Compound 6] | % Inhibition | |
| --- | --- | --- |
| -4.0 | 95.0 | 94.0 |
| -5.0 | 80.0 | 79.0 |
| -6.0 | 22.0 | 21.0 |
| -7.0 | 2.0 | 0.0 |
| -8.0 | -8.0 | -11.0 |
| -9.0 | -1.0 | -3.0 |
| -10.0 | -11.0 | -10.0 |

**Figure 5**

| Species | Sample Name | 2-NT | 3-NT | 4-NT | 5-NT | 6-NT | 7-TNF-a | 8-TNF-a | 9-TNF-a | 10-TNF-a | 11-TNF-a | 12-TNF-a |
| --- | --- | --- | --- | --- | --- | --- | --- | --- | --- | --- | --- | --- |
| d18:1/16:0 | | 1.666144 | 1.5032 | 1.432287 | 1.855049 | 1.799714 | 2.181321 | 2.470174 | 2.214855 | 2.626016 | 2.723684 | 1.914312 |
| d18:1/18:0 | | 6.316614 | 5.892961 | 4.612856 | 6.541802 | 6.862184 | 8.892201 | 9.600752 | 8.529412 | 9.561879 | 9.736842 | 7.684594 |
| d18:1/20:0 | | 0.398851 | 0.279988 | 0.319235 | 0.478773 | 0.420744 | 0.603577 | 0.612494 | 0.668495 | 0.656278 | 0.629049 | 0.493163 |
| d18:1/22:0 | | 0.152194 | 0.16178 | 0.106379 | 0.186482 | 0.155412 | 0.213015 | 0.219634 | 0.197807 | 0.203433 | 0.217409 | 0.179307 |
| d18:1/24:0 | | 0.189707 | 0.218499 | 0.144504 | 0.184799 | 0.182976 | 0.205912 | 0.201127 | 0.231157 | 0.270506 | 0.242713 | 0.200228 |
| d18:1/24:1 | | 0.14582 | 0.107155 | 0.102552 | 0.161238 | 0.1268 | 0.247193 | 0.252889 | 0.28659 | 0.293406 | 0.277328 | 0.184959 |
| ***Dihydroceramides*** | |  |  |  |  |  |  |  |  |  |  |  |
| d18:0/18:0 | | 0.092111 | 0.09587 | 0.069087 | 0.118295 | 0.092036 | 0.135966 | 0.143495 | 0.123629 | 0.155239 | 0.144585 | 0.135278 |
| ***Monohexosylceramides*** | | |  |  |  |  |  |  |  |  |  |  |
| d18:1/24:1 | | 3.75E-02 | 3.09E-02 | 3.00E-02 | 7.12E-02 | 3.74E-02 | 6.42E-02 | 8.57E-02 | 6.27E-02 | 9.28E-02 | 9.45E-02 | 8.80E-02 |
| d18:1/16:0 | | 8.24E-02 | 5.75E-02 | 7.52E-02 | 1.00E-01 | 8.70E-02 | 1.25E-01 | 1.46E-01 | 1.28E-01 | 1.65E-01 | 1.78E-01 | 1.43E-01 |
| d18:1/18:0 | | 9.07E-01 | 6.51E-01 | 7.41E-01 | 7.09E-01 | 8.34E-01 | 8.09E-01 | 8.11E-01 | 9.45E-01 | 8.33E-01 | 9.27E-01 | 8.86E-01 |
| d18:1/20:0 | | 1.05E-01 | 5.36E-02 | 7.75E-02 | 8.45E-02 | 9.70E-02 | 1.34E-01 | 1.36E-01 | 1.34E-01 | 1.68E-01 | 1.71E-01 | 1.46E-01 |
| d18:1/22:0 | | 3.73E-02 | 3.02E-02 | 4.26E-02 | 5.09E-02 | 5.35E-02 | 5.38E-02 | 5.08E-02 | 5.16E-02 | 6.26E-02 | 5.07E-02 | 4.08E-02 |
| d18:1/24:0 | | 4.86E-02 | 5.46E-02 | 2.56E-02 | 4.71E-02 | 3.84E-02 | 3.48E-02 | 3.50E-02 | 3.93E-02 | 4.46E-02 | 4.59E-02 | 3.01E-02 |
| ***DihydroGlc*** | |  |  |  |  |  |  |  |  |  |  |  |
| d18:0/18:0 | | 0.028234 | 0.013915 | 0.018989 | 0.015869 | 0.018097 | 0.020248 | 0.027581 | 0.011396 | 0.013898 | 0.024211 | 0.021399 |
| ***Dihexosylceramides*** | | |  |  |  |  |  |  |  |  |  |  |
| d18:1/16:0 | | 0.018239 | 0.018842 | 0.020746 | 0.02019 | 0.014683 | 0.016071 | 0.029258 | 0.02318 | 0.021197 | 0.026184 | 0.029075 |
| d18:1/18:0 | | 0.095298 | 0.066259 | 0.057605 | 0.077579 | 0.069289 | 0.097864 | 0.133631 | 0.132004 | 0.130714 | 0.153441 | 0.115087 |

| Species | Sample Name | 13-Cam | 14-Cam | 15-Cam | 16-Cam | 17-Cam | 18-Cam | 19-Cam+TNF-a | 21-Cam+TNF-a | 22-Cam+TNF-a | 23-Cam+TNF-a | 24-Cam+TNF-a |
| --- | --- | --- | --- | --- | --- | --- | --- | --- | --- | --- | --- | --- |
| d18:1/16:0 | | 1.521025 | 1.884298 | 1.702015 | 1.620008 | 1.612973 | 1.723467 | 1.410437 | 2.429479 | 1.855086 | 2.133222 | 1.954374 |
| d18:1/18:0 | | 5.804737 | 7.280992 | 7.293666 | 5.792803 | 5.652921 | 6.477801 | 5.880582 | 9.495976 | 6.314779 | 7.764363 | 7.043741 |
| d18:1/20:0 | | 0.4116 | 0.516529 | 0.460557 | 0.421115 | 0.480241 | 0.498097 | 0.391069 | 0.481576 | 0.488964 | 0.549542 | 0.527526 |
| d18:1/22:0 | | 0.123296 | 0.163347 | 0.160029 | 0.119098 | 0.144588 | 0.146385 | 0.104917 | 0.152308 | 0.147745 | 0.184055 | 0.145023 |
| d18:1/24:0 | | 0.168246 | 0.127975 | 0.15691 | 0.13013 | 0.147036 | 0.152812 | 0.158705 | 0.169166 | 0.156814 | 0.203206 | 0.167006 |
| d18:1/24:1 | | 0.122523 | 0.204959 | 0.172169 | 0.118703 | 0.202448 | 0.194376 | 0.142449 | 0.284752 | 0.151248 | 0.212781 | 0.183861 |
| ***Dihydroceramides*** | |  |  |  |  |  |  |  |  |  |  |  |
| d18:0/18:0 | | 0.09884 | 0.111488 | 0.109357 | 0.092487 | 0.094759 | 0.095476 | 0.088409 | 0.132571 | 0.095681 | 0.139384 | 0.099623 |
| ***Monohexosylceramides*** | | |  |  |  |  |  |  |  |  |  |  |
| d18:1/24:1 | | 4.71E-02 | 5.24E-02 | 4.29E-02 | 4.22E-02 | 5.04E-02 | 4.78E-02 | 5.62E-02 | 1.19E-01 | 8.03E-02 | 5.61E-02 | 4.36E-02 |
| d18:1/16:0 | | 1.02E-01 | 1.18E-01 | 9.48E-02 | 1.13E-01 | 1.03E-01 | 1.09E-01 | 9.20E-02 | 2.24E-01 | 1.17E-01 | 1.16E-01 | 1.22E-01 |
| d18:1/18:0 | | 8.46E-01 | 7.75E-01 | 8.00E-01 | 6.92E-01 | 7.61E-01 | 8.63E-01 | 7.79E-01 | 9.48E-01 | 7.24E-01 | 8.33E-01 | 6.89E-01 |
| d18:1/20:0 | | 1.06E-01 | 1.36E-01 | 1.28E-01 | 1.12E-01 | 1.09E-01 | 1.15E-01 | 1.19E-01 | 1.74E-01 | 1.20E-01 | 1.32E-01 | 1.35E-01 |
| d18:1/22:0 | | 5.44E-02 | 4.39E-02 | 4.15E-02 | 4.38E-02 | 3.58E-02 | 4.79E-02 | 3.73E-02 | 5.82E-02 | 5.01E-02 | 4.50E-02 | 5.26E-02 |
| d18:1/24:0 | | 3.38E-02 | 3.70E-02 | 4.37E-02 | 2.84E-02 | 2.01E-02 | 3.57E-02 | 2.51E-02 | 4.11E-02 | 3.36E-02 | 4.30E-02 | 4.11E-02 |
| ***DihydroGlc*** | |  |  |  |  |  |  |  |  |  |  |  |
| d18:0/18:0 | | 0.009797 | 0.017198 | 0.019976 | 0.017513 | 0.012556 | 0.017873 | 0.015424 | 0.018759 | 0.012644 | 0.023963 | 0.012029 |
| ***Dihexosylceramides*** | | |  |  |  |  |  |  |  |  |  |  |
| d18:1/16:0 | | 0.016501 | 0.026719 | 0.030715 | 0.020668 | 0.023041 | 0.027945 | 0.024446 | 0.04388 | 0.020821 | 0.036503 | 0.026056 |
| d18:1/18:0 | | 0.074529 | 0.117273 | 0.095202 | 0.101582 | 0.090034 | 0.108626 | 0.083894 | 0.171283 | 0.091795 | 0.116611 | 0.11135 |

**Figure 6A, left (IL1β)**

| No IL1β | | | 1 0 0 n g / m l I L 1 β | | | | | | | | |
| --- | --- | --- | --- | --- | --- | --- | --- | --- | --- | --- | --- |
| 25 μM Cambinol | | | No compound | | | 0.1 μM Cambinol | | | 0.3 μM Cambinol | | |
| Mean | SD | N | Mean | SD | N | Mean | SD | N | Mean | SD | N |
| 91.20892 | 6.016893 | 18 | 70.307830 | 11.860320 | 19 | 77.246780 | 10.693110 | 8 | 74.025160 | 8.616473 | 15 |

Continued…

| 1 0 0 n g / m l I L 1 β | | | | | | | | | | | |
| --- | --- | --- | --- | --- | --- | --- | --- | --- | --- | --- | --- |
| 1 μM Cambinol | | | 3 μM Cambinol | | | 10 μM Cambinol | | | 25 μM Cambinol | | |
| Mean | SD | N | Mean | SD | N | Mean | SD | N | Mean | SD | N |
| 84.507760 | 7.677381 | 15 | 93.006200 | 8.459284 | 12 | 93.583580 | 6.691028 | 9 | 97.122570 | 7.549228 | 11 |

Continued…

| 1 0 0 n g / m l I L 1 β | | | | | | | | | | | |
| --- | --- | --- | --- | --- | --- | --- | --- | --- | --- | --- | --- |
| 10 μM Compound 2 | | | 25 μM Compound 2 | | | 10 μM Zoledronic acid | | | 25 μM Zoledronic acid | | |
| Mean | SD | N | Mean | SD | N | Mean | SD | N | Mean | SD | N |
| 79.083330 | 9.323750 | 22 | 81.702800 | 11.632490 | 24 | 84.876100 | 8.801410 | 22 | 85.752590 | 10.176970 | 27 |

**Figure 6A, right (TNFα)**

| 1 0 0 n g / m l T N F α | | | | | | | | | | | |
| --- | --- | --- | --- | --- | --- | --- | --- | --- | --- | --- | --- |
| No compound | | | 0.1 μM Cambinol | | | 0.3 μM Cambinol | | | 1 μM Cambinol | | |
| Mean | SD | N | Mean | SD | N | Mean | SD | N | Mean | SD | N |
| 66.934300 | 10.285400 | 9 | 78.254980 | 5.465128 | 12 | 82.519750 | 9.034270 | 11 | 86.256630 | 7.077134 | 13 |

Continued…

| 1 0 0 n g / m l T N F α | | | | | | | | |
| --- | --- | --- | --- | --- | --- | --- | --- | --- |
| 3 μM Cambinol | | | 10 μM Cambinol | | | 25 μM Cambinol | | |
| Mean | SD | N | Mean | SD | N | Mean | SD | N |
| 90.168630 | 6.875567 | 14 | 91.481070 | 8.211434 | 7 | 92.363970 | 7.099655 | 7 |

Continued…

| 1 0 0 n g / m l T N F α | | | | | | | | | | | |
| --- | --- | --- | --- | --- | --- | --- | --- | --- | --- | --- | --- |
| 10 μM Compound 2 | | | 25 μM Compound 2 | | | 10 μM Zoledronic acid | | | 25 μM Zoledronic acid | | |
| Mean | SD | N | Mean | SD | N | Mean | SD | N | Mean | SD | N |
| 76.551190 | 10.958210 | 27 | 81.796990 | 10.033960 | 27 | 80.065990 | 9.685423 | 26 | 81.276550 | 7.718798 | 28 |

**Figure 6B**

| N o T N F α | | | | | | | | |
| --- | --- | --- | --- | --- | --- | --- | --- | --- |
| No compound | | | 200 nM CHIC-35 | | | 40 μM sirtinol | | |
| Mean | SD | N | Mean | SD | N | Mean | SD | N |
| 99.95923 | 6.452137 | 27 | 62.07029 | 7.57668 | 15 | 53.29902 | 6.847779 | 19 |

| 1 0 0 n g / m l T N F α | | | | | | | | |
| --- | --- | --- | --- | --- | --- | --- | --- | --- |
| No compound | | | 200 nM CHIC-35 | | | 40 μM sirtinol | | |
| Mean | SD | N | Mean | SD | N | Mean | SD | N |
| 72.51649 | 10.95821 | 13 | 71.87894 | 9.172855 | 17 | 60.72948 | 12.97132 | 17 |

**Figure 7**

| No TNFα | | | | | |
| --- | --- | --- | --- | --- | --- |
| Control | | | 30 μM Cambinol | | |
| Mean | SD | N | Mean | SD | N |
| 16010.0 | 4648.096 | 87 | 15151.25 | 6266.454 | 125 |

| 1 0 0 n g / m l T N F α | | | | | | | | | | | |
| --- | --- | --- | --- | --- | --- | --- | --- | --- | --- | --- | --- |
| No compound | | | 0.1 μM Cambinol | | | 0.3 μM Cambinol | | | 1 μM Cambinol | | |
| Mean | SD | N | Mean | SD | N | Mean | SD | N | Mean | SD | N |
| 9861.429 | 4686.798 | 83 | 9111.111 | 3015.744 | 129 | 11450 | 4580.005 | 116 | 13799 | 5139.686 | 117 |

| 1 0 0 n g / m l T N F α | | | | | | | | |
| --- | --- | --- | --- | --- | --- | --- | --- | --- |
| 3 μM Cambinol | | | 10 μM Cambinol | | | 30 μM Cambinol | | |
| Mean | SD | N | Mean | SD | N | Mean | SD | N |
| 14262.5 | 2697.585 | 124 | 18867 | 6534.014 | 138 | 16000 | 4991.421 | 99 |
