# Supplementary material for: Cambinol, a Novel Inhibitor of Neutral Sphingomyelinase 2 Shows Neuroprotective Properties
Source: PLoS One. 2015 May 26;10(5):e0124481. doi: 10.1371/journal.pone.0124481 (PMC4444023; doi:10.1371/journal.pone.0124481)
Supplement: S1 Table — (DOCX) [file pone.0124481.s007.docx]

**Table S1: Cambinol inhibitory activity against human nSMase2 lacks time dependence.**

| **Incubation time (min)^a^** | **IC_50_ (μM)^b^** |
| --- | --- |
| 0 | 12 |
| 1 | 11 |
| 15 | 9 |
| 30 | 8 |
| 60 | 8 |
| 120 | 5 |

^a^For cambinol and human nSMase2 prior to initiation of the reaction.

^b^Cambinol IC_50_ values were determined from 8-points dose response curves run in duplicate.
